# Piloting an ICU follow-up clinic — qualitative process evaluation alongside a feasibility study

**DOI:** 10.1186/s40814-026-01827-5

**Published:** 2026-05-09

**Authors:** Karl Philipp Drewitz, Christine Bernardi, Katharina Pielmeier, Susanne Brandstetter, Magdalena Rohr, Vreni Brunnthaler, Christoph Fisser, Maximilian V. Malfertheiner, Christian Apfelbacher

**Affiliations:** 1 Institute of Social Medicine and Health Systems Research, Otto Von Guericke University Magdeburg, Medical Faculty, Leipziger Str. 44, Magdeburg, 39120 Germany; 2 Medical Sociology, Institute of Epidemiology and Preventive Medicine, University of Regensburg, Dr.-Gessler-Str. 17, Regensburg, 93051 Germany; 3 Department of Internal Medicine I, University Hospital Regensburg, Franz-Josef-Strauss-Allee 11, Regensburg, 93053 Germany; 4 University Children’s Hospital Regensburg (KUNO-Clinics), University of Regensburg, Klinik St. Hedwig, Steinmetzstr. 1-3, Regensburg, 93049 Germany; 5 Caritas-Krankenhaus St. Josef, Landshuter Str. 65, Regensburg, 93053 Germany; 6 Department of Internal Medicine II, University Hospital Regensburg, Franz-Josef-Strauss-Allee 11, Regensburg, 93053 Germany; 7 Caritas-Krankenhaus St. Maria, Ludwigstr. 68, Donaustauf, 93093 Germany

**Keywords:** ICU follow-up, ICU patients, Qualitative process evaluation

## Abstract

**Background:**

Previous studies have not demonstrated sufficient effects of intensive care unit (ICU) follow-up clinics on health-related quality of life. This may also be influenced by the design and implementation of these studies. Feasibility and context of such studies have yet to be sufficiently researched. It is therefore crucial to investigate these contextual factors. This study aimed to explore the experiences of patients, relatives, and healthcare professionals with both the randomised controlled trial (RCT) and the ICU follow-up clinic, to assess the feasibility and acceptability of the intervention and the study design from the perspectives of those involved.

**Methods:**

This qualitative research was embedded in the process evaluation of a pilot RCT on an ICU follow-up clinic. We conducted 18 semi-structured interviews with former ICU patients, relatives and the clinical study team who participated in the PINA study and used field notes from the clinical study team. Data analysis was carried out using qualitative content analysis.

**Results:**

The pilot RCT was well received by potential patients and their relatives, and the motivation to participate was high, as was the motivation of the clinical study team. The main benefits of the ICU follow-up clinic intervention named by participants were being taken seriously and receiving referrals that they would not typically get from a general practitioner. The offer of home visits for patients with long travel distances to the ICU follow-up clinic was highly appreciated.

**Conclusions:**

It was feasible to integrate the perspectives of patients, relatives, and healthcare professionals within a pilot RCT on an ICU follow-up clinic. Across all stakeholder groups, the ICU follow-up clinic and the RCT trial design were considered acceptable and valuable, suggesting that such follow-up care meets patient needs and that a large-scale study appears feasible.

**Trial registration:**

ClinicalTrials.gov US NLM, NCT04186468, Submission: 02/12/2019, Registration: 04/12/2019, https://clinicaltrials.gov/ct2/show/NCT04186468.

**Supplementary Information:**

The online version contains supplementary material available at 10.1186/s40814-026-01827-5.

## Key messages regarding feasibility

- What uncertainties existed regarding the feasibility?
  There is uncertainty regarding feasibility, patient and caregiver acceptance of and actual attendance at ICU follow-up clinics.
  Staffing, scheduling, and other logistical hurdles are not yet sufficiently documented, requiring qualitative process evaluation to understand implementation barriers.
  Lack of consensus on efficacy and outcome measures limits demonstration of benefits, underscoring the need for feasibility studies on acceptability, integration, and evaluation frameworks.
- What are the key feasibility findings?
  The pilot RCT and ICU follow-up clinic achieved high participation and acceptance among patients, caregivers and the study team.
  Participants valued feeling taken seriously and receiving referrals beyond standard GP care and highlighted physical and psychosocial benefits.
  Some participants in the control group perceived follow-up surveys as an intervention and challenges emerged around poor recall of informed consent.
- What are the implications of the feasibility findings for the design of the main study?
  High participation and acceptance support the general feasibility of a larger RCT on the effects of an ICU follow-up clinic.
  Eligibility criteria, communication on the study while recruiting participants, particular survey design and selection of measurement instruments should be considered once again before conducting the definitive trial.

## Background

The number of intensive care treatment cases in Germany is constantly high [1–3]. Due to medical progress, the survival rate for patients treated in an intensive care unit (ICU) has risen in industrialised nations [4]. However, for several patients the recovery process after a critical illness is long and resource intensive [5]. Many survivors suffer from physical, cognitive and mental impairments after discharge from the ICU. These symptoms and disorders are known as post-intensive care syndrome (PICS) [4, 6]. Even if epidemiological data from international studies on PICS are scarce, it is assumed that at least one-half of ICU survivors suffer from at least one component of PICS after discharge from the ICU [7–10].

It has long been known that it is not only the mere survival of an ICU stay that is decisive, but also the health-related quality of life (HRQOL) afterwards [11–13]. Over the past 40 years it has become apparent that the consequences of the ICU stay—nowadays summarised under PICS—are usually associated with a lower health-related quality of life among ICU survivors [14–19]. Thus, some countries already offer ICU follow-up services, especially the UK, the USA and Scandinavian countries [20–23]. Several ICU follow-up models have already been evaluated and, even if they had no statistically significant effect on the health-related quality of life (HRQOL) [20], qualitative studies could nevertheless show a benefit, particularly in the psychosocial domain [24–27]. Furthermore, caregivers have also described post-ICU follow-up care as beneficial [28].

In Germany, to the best of our knowledge, there is a lack of innovative care models, which assist patients after discharge from the ICU along the care pathway. However, the demand for structured aftercare remains high [29, 30].

The German health care system is characterised by a strong institutional fragmentation, impeding seamless patient transitions, particularly after ICU care [31]. This division arises from multiple financing bodies—statutory health insurers, pension and accident insurance providers, private insurers, and public payers—each with distinct reimbursement systems and IT infrastructures [32]. As a result, provider coordination is limited, causing discontinuity of care, redundant diagnostics, and suboptimal outcomes during transfers between inpatient, primary, specialist, and rehabilitation services. ICU follow-up services are unevenly available and face organisational and structural barriers, which complicate timely continuity of care after critical illness [33, 34]. Most rehabilitation services in Germany are discipline-specific and focus on particular organ failures rather than catering to the broader needs of former ICU patients [35]. Typically, former ICU patients with severe underlying conditions are referred to specialised rehabilitation facilities for early intervention, tailored to their individual requirements. Conversely, follow-up rehabilitation is provided when acute medical treatment is no longer necessary and patients can perform daily tasks independently [36]. However, the transition from rehabilitation to ongoing care often leaves a gap in support, particularly for patients with complex underlying conditions. This highlights the need for specialised follow-up services to ensure optimal care and outcomes for these patients.

Therefore, we developed a concept for an ICU follow-up clinic in a participatory process [37–39], which involved interviews with former ICU patients and their relatives as well as focus group discussions, expert interviews and workshops including a total of nine different professions. The intervention consisted of three main components: information (e.g. information pamphlet for patients and relatives), consultation (e.g. visit to the ICU follow-up clinic), and networking (e.g. special referral letter). The ICU follow-up clinic was pilot tested in a pragmatic randomised controlled trial (RCT), with the following feasibility domains and outcomes: acceptability (consent rate, acceptance of randomisation), fidelity (intervention delivery), attrition rate, completeness of measurement instruments and practicality (number of participants requiring assistance with questionnaires). The feasibility study showed strong acceptability, with 85% of eligible approached patients consenting and 100% of those enrolled accepting their randomised allocation. Fidelity was moderate: 62% of participants in the intervention arm received every protocol component. Completeness was mixed: attrition reached 34%, yet baseline instruments were fully completed (100%) and follow-up outcome measures were 77% complete on average. Practicality indicated a growing respondent burden, as questionnaire assistance was used by 27% of participants at baseline but rose to 85% at follow‑up [40].

Part of the feasibility assessment was also a qualitative process evaluation to further examine the feasibility, challenges and supporting factors for the ICU follow-up clinic and a larger effectiveness trial. Healthcare interventions targeting complex health challenges, such as those following ICU treatment, typically involve multifaceted approaches that interact with complex systems. The success of these interventions often relies on the interplay between various components and their adaptability to specific contexts [41]. Consequently, understanding the processes that shape the development and delivery of these interventions is crucial for their success. Studies conducted to date on ICU follow-up services show a high degree of variability in terms of the components of the interventions [42, 43]. Furthermore, these studies have often failed to adequately examine the context of these complex interventions (e.g. quite fundamentally the acceptability to clinicians) and subsequent implementability [43]. Purely quantitative methods often struggle to capture the context in this regard. Qualitative process evaluation emerges as a useful component of feasibility assessment, providing insights into the nuanced processes that underlie the complex healthcare intervention in question. One of the key strengths of qualitative process evaluation is its emphasis on the contextual factors influencing intervention implementation [44].

Against this background, the aim of this study was to substantiate the quantitative elements of the feasibility study and to assess feasibility, acceptability and the level of satisfaction among participants (patients, relatives and the clinical study team) with the pilot RCT itself, the procedures, the ICU follow-up clinic and the context of the pilot RCT on a qualitative perspective.

## Methods

### Setting and study design

This qualitative process evaluation study was part of a pilot RCT on an ICU follow-up clinic [40]. In this study, feasibility of the pilot RCT and its procedures as well as the feasibility of the intervention itself was covered.

The pilot RCT included 41 participants from three ICUs (two medical and one surgical ICU) at the University Hospital Regensburg, Germany. Participants had to meet following inclusion criteria: 18 years or older, written informed consent, duration of ICU stay more than 5 days, SOFA (sequential organ failure assessment)-score greater than five at any time of the ICU stay and expected survival time greater than 6 months estimated by intensivists and were randomised 1:1. The intervention group received several intervention components, e.g. an information pamphlet for patients and relatives, a telephone monitoring, a visit of the ICU follow-up clinic 11–12 weeks after discharge and a referral letter of the ICU follow-up clinic. The control group received usual care [40, 45]. The main effectiveness outcome of the pilot RCT was the assessment of physical HRQOL 6 months after inclusion into the study. This was evaluated using the Physical Component Scale (PCS) of the Short Form-12 (SF-12) self-report questionnaire, which reflects a 4-week recall period [46]. Secondary outcomes included measures of physical functioning (chair rising test and hand grip strength assessment), activities of daily living (Barthel index), mental and social impairments (PTSS-10 and PHQ-9 questionnaire), as well as the frequency of ambulatory and inpatient healthcare utilisation post-ICU discharge. Additionally, the HRQOL of the next of kin was evaluated (SF-12).

Further, the clinical study team, composed of two clinicians and two study nurses, also participated as study participants in the qualitative process evaluation and took part in interviews. All interviewees provided written informed consent prior to participation. Following the MRC framework [47–49], we aimed to explore feasibility and acceptability of the pilot RCT and the ICU follow-up clinic with a mixed-methods process evaluation [50]. We developed a logic model to structure and contextualise the process evaluation, see Fig. 5 at Rohr et al. [45], which also guided the research questions for the qualitative process evaluation. As part of the quantitative evaluation, we defined acceptability, fidelity, completeness and practicality as domains of feasibility [40]. Regarding the qualitative process evaluation, we designed a qualitative, explorative study and conducted semi-structured interviews with open-ended questions to explore ICU survivors’, relatives’ and healthcare professionals’ experience with the pilot RCT, its context and the ICU follow-up clinic. We aimed to interview 10 study participants in the intervention and control groups, respectively [45]. MR and VB developed interview guides (see Supplementary material 1). We discussed and refined the interview guides in the research team after the first interviews. In particular, the interview guides covered the acceptance of individual steps in the implementation of the study, e.g. information flow, consent, scope of the questionnaires, concerns and individual challenges in participation. With regard to the ICU follow-up clinic, we were mainly interested in acceptance, feasibility of individual components.

To record additional contextual factors, one recruitment interview, one ICU follow-up clinic visit and one follow-up examination were observed by study team members (VB and CB).

Further, we instructed the clinical study team to take field notes to identify factors that might hinder or improve the feasibility of the pilot RCT, or even provide guidance for the implementation of the ICU follow-up clinic in everyday care.

### Participants

We reached out to every participant of the pilot RCT, who was available for the 6-month follow-up (27/41). Potential participants had to be characterised by having no serious cognitive impairments and sufficient knowledge of German. Information material and informed consent forms were distributed and some participants signed right away. Other participants took them home and sent them back to the study team by mail. Furthermore, we interviewed the healthcare professionals who were working at the ICU follow-up clinic and formed the clinical study team.

### Data collection and analysis

Between January 2021 and April 2021, after completion of the pilot RCT, a researcher (MSc) with an ICU nursing background, interested in this field and with prior experience in qualitative research (CB) conducted 18 single individual telephone interviews with nine survivors, five relatives and four healthcare professionals (see Table 1). The interviewer had no prior relationship with the patients and relatives but knew the healthcare professionals. The duration of the interviews ranged from 11 to 60 min (median 22 min). Written informed consent was obtained in advance. At the beginning, participants were again informed about the purpose of the interview study. The interviewees were told that they might omit a question or end the interview at any time if they felt uncomfortable. We conducted the interviews in German and took field notes after each interview in addition to the audio recording.

**Table 1:** Characteristics of the interview participants

Interview ID	Group	Additional characteristics
I001	Relative, intervention group	Male, 74 years
I002	Patient, intervention group	Female, 74 years
I003	Patient, intervention group	Male, 54 years
I004	Relative, intervention group	Female, 53 years
I005	Relative, intervention group	Female, 65 years
I006	Patient, intervention group	Male, 69 years
I007	Patient, intervention group	Male, 66 years
I008	Relative, intervention group	Female, 66 years
K001	Patient, control group	Male, 72 years
K002	Patient, control group	Female, 65 years
K003	Patient, control group	Female, 76 years
K004	Patient, control group	Male, 56 years
K005	Patient, control group	Male, 45 years
K006	Relative, control group	Female, 42 years
E001	Study nurse	
E002	Clinician, study physician	
E003	Study nurse	
E004	Clinician, study physician	

The interviews were audio recorded and transcribed verbatim. Data were processed computer-assisted with the QDA (qualitative data analysis) software ATLAS.ti [51]. Data analysis was carried out using qualitative content analysis [52]. The systematic approach [53] used in qualitative content analysis enabled us to identify common topics in patients’, relatives’ and health professionals’ interviews. Five interviews were double-coded by KP to check the completeness and accuracy of the coding framework. The first step in data analysis was a deductive approach. A category system was created by extracting thematic blocks from the interview guide. This determined the substantive aspects to be filtered out of the material. In a second step, further categorisation took place in the form of an inductive formation of additional categories. The categories were used to create a system for interpreting the content of the texts. The category system was then discussed in the team before the results were summarised. Transcripts or summarised findings were not shared with participants.

### Ethical and privacy considerations

The ethics committee of the University of Regensburg approved the study on 26/09/2019 (19-1522-101). Prior to the start of the interview, the interviewer verified again that written informed consent for the study had been given. Every participant actively agreed to be part of the study and informed consent was obtained according to the Helsinki Declaration. Personal information was only collected on the consent form and stored separately from study data. Transcripts of the interviews were de-identified (pseudonymised). The feasibility study was registered at ClinicalTrials.gov (NCT04186468). Consent to publish the results of the interviews with the study physicians and nurses has been obtained.

## Findings

**Fig. 1:**
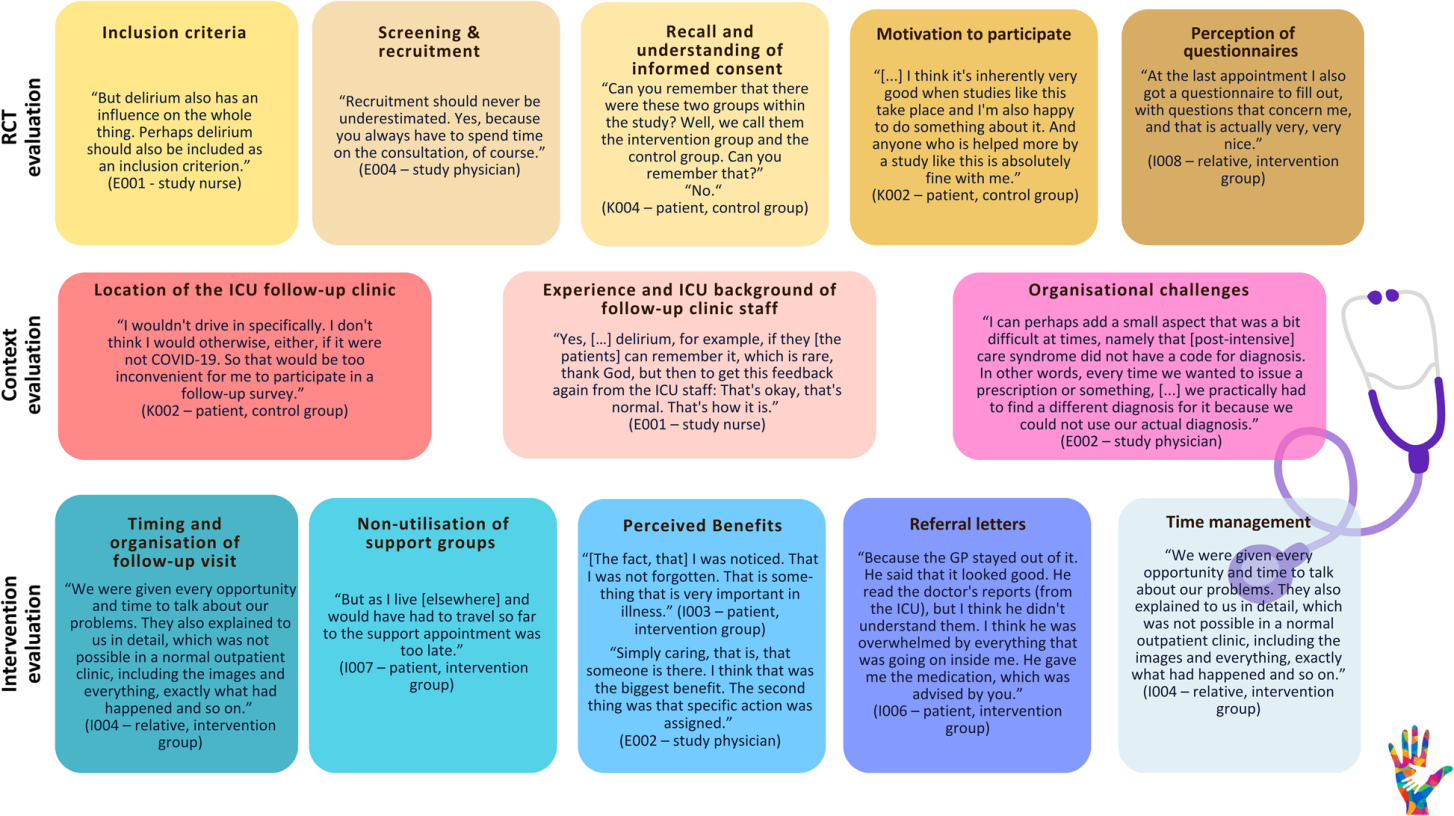
Main themes illustrated with representative quotes

The identified main themes illustrated with representative quotes can be found in Fig. 1.

### Evaluation of the pilot randomised controlled trial (RCT)

#### Suitability of the inclusion criteria

With regard to the inclusion and exclusion criteria, suggestions for changes were made from the clinical-study team. One person suggested delirium as an additional inclusion criterion. Another person suggested not to include patients after organ transplantation or actively undergoing cancer treatment because they already receive close follow-up services. For one interview participant, a duration of ventilation of more than 5 days should become an inclusion criterion.

“But delirium also has an influence on the whole thing. Perhaps delirium should also be included as an inclusion criterion.” (E001—study nurse)

“There are also patients who are well cared for, even without the ICU follow-up clinic, i.e. oncological patients, transplant recipients or candidates who are still waiting for a transplant.” (E003—study nurse)

“I would perhaps rather say, if I were doing a larger study, people who were ventilated for at least five days. And then say, well, organ transplants are out, oncological patients, in other words patients with major oncological operations are out.” (E004—study physician)

Relatives were not in the focus of the ICU follow-up clinic. One interviewee suggested addressing them separately in a large-scale study.

“The relatives definitely fell short. Personally, I would have been interested: How are they doing? Also, maybe having a separate conversation. Because they could remember everything that happened in the ICU.” (E001—study nurse)

#### Screening and recruitment of the study participants

Patient screening for study inclusion was considered well structured, but more time-consuming than planned. As part of our observation (field notes), we found that patient screening took 1–2 h a day during the recruitment period (3 months). It was only possible because extra staff (two study nurses) were assigned to this task. The study team reflected that screening and recruitment efforts were different when the pilot RCT was restarted after a COVID break, as there was significantly less time available for this step due to the project duration specified by the funding organisation. The patient profile was different as patients on one ward did not meet inclusion criteria since they were often already too well (field notes). Further, the assessment of eligibility of a patient using information from the electronic records alone was often not sufficient.

“And it varied, because some of them may have met all the criteria and were potentially eligible for inclusion. But when you were on site, you found out: Okay, there’s another big surgery coming up next week, where he’s going to be intubated again anyway, so we’ll still have to move him, so I’d say that of those who were potentially suitable for being included, we were probably able to include a third of them directly and the majority we then had to postpone or it turned out that he might not be suitable for inclusion yet.” (E002—study physician)

The clinical study team further reflected that for the planning of a large-scale effectiveness trial, the time required should clearly be taken into account.

“Recruitment should never be underestimated. Yes, because you always have to spend time on the consultation, of course.” (E004—study physician)

#### Recall and understanding of informed consent conversation

The patients and relatives were mostly satisfied with how and how much information they received about the pilot RCT during the informed-consent conversation.

“I say it’s tailored to me so that you can understand it. And not be overwhelmed with foreign words. No, it was just right.” (I003—patient, intervention group)

“Well, I was able to ask questions if I didn’t understand something. And that was actually good. I was informed and understood it. And I liked it.” (I001—relative, intervention group)

“How can I put it, we basically got answers to our questions in the [hospital], or rather I did, when my husband was still in a different condition and in a coma. So I was very well informed and I didn’t feel any deficits in this respect.” (I005—relative, intervention group)

Although they felt well informed, several patients and some relatives could not remember or recall the informed consent conversation. At the time of the interview, very few interviewees could recall the information about randomisation and knew which group they were assigned to.

“Can you still remember the explanation, i.e. when the doctor was with you in the intensive care unit and explained the study to you?”

“Honestly, nothing. [...]. Well, I have to be honest, I was transferred [...] and I don’t really have many memories [...]. Now I have to ask my wife directly whether she noticed anything.”

Wife of participant: “Yes, we signed something, but I can’t remember exactly what it was because I was, well, very excited at the time.” (K001—patient, control group)

“Can you remember that there were these two groups within the study? Well, we call them the intervention group and the control group. Can you remember that?”

“No.” (K004—patient, control group)

“There were some patients who couldn’t really remember anything at that stage. So that would also be something where you could say: Okay, it would be important to talk to the relatives. So that they understand: What is this about? And that they can later explain to the patients what an ICU follow-up clinic is. That was also something I noticed. Some were just too weak and didn’t realize it at all.” (E001—study nurse)

One reason might be that the informed consent conversation took place too early. At the time of discharge from ICU, they felt not yet able to understand the entire extent of the pilot RCT.

“It was very good, but too early. [So I am] sticking to that. A bit too early. I would have waited another day, maybe two days.” (I001—patient, intervention group)

Only one person was therefore able to comment in more detail on the acceptance of randomisation.

“As you know, this study had two groups. [...]. Did you realize that?”

“Yes.”

“How was that for you? Well, there was also the possibility that your husband would be in the other group and then get nothing, so to speak.”

“Yes. Well, I said that we’re lucky now that we’re actually in the active group or actively supervised group.” (I005—relative, intervention group)

#### Motivation to participate in the pilot RCT

The interviewees in the qualitative study expressed a generally high motivation to participate in the RCT. They also mentioned the relevance of the trial for future ICU patients.

“[...] I think it’s inherently very good when studies like this take place and I’m also happy to do something about it. And anyone who is helped more by a study like this is absolutely fine with me.” (K002—patient, control group)

“So, I think it’s very good that you do something like this and that you can perhaps help other people, too.” (I008—relative, intervention group)

This is reflected in health professionals’ accounts:

“I found the willingness of the patients extremely high. So you really saw that this is a topic that is important for patients. Having so few patients in a study who say “No, I’m not taking part”, is rare.” (E004—study physician)

Regarding their own motivation, the health professionals focused on the relevance of follow-up services for former ICU patients. This relevance was not only recognised in the clinical study team, but also in other units of the hospital apart from the ICU.

“Everyone in the healthcare system actually says “hey, that’s great, we need that (ICU follow-up)” and that’s rare that you have, let’s say, overall positive feedback from all sides.” (E004—study physician)

#### Perception of the questionnaire completion

When asked specifically about the extent of the questionnaires, we received different answers regarding the follow-up survey.

“There were a lot of questions at first, so you think: “Oh God, oh God, oh God.” But once you got into the flow, let’s say, it actually worked. Well, for me at least.” (K004—patient, control group)

Interviewer: “We talked earlier about the questionnaires. What do you think about them?” “They were simple and reasonable.” (K001—patient, control group)

“Yes, that was definitely too much. Yes, and the questions, especially the questions, were repeated over and over again.” (K006—relative, control group)

Interviewer: “Do you remember the questionnaires?” “I remember them [...], because I also have difficulty reading, which means [...] I sometimes have to read something twice.” (K002—patient, control group)

Others appreciated receiving and filling out questionnaires—they perceived this as a form of attention, even in the control group or among the relatives.

“It’s clear, I mean, the questionnaire just tells me, ok, they want to know how I’m doing and it’s not finished with the discharge from the hospital.” (I002—patient, intervention group)

“At the last appointment I also got a questionnaire to fill out, with questions that concern me, and that is actually very, very nice.” (I008—relative, intervention group)

“You then realize that the patient is still important afterwards. I think that’s a good thing about the study, because normally you are discharged from hospital and then it’s over.” (K001—patient, control group)

Both study nurses mentioned that participants completed questionnaires with their assistance in some instances, which was time-consuming on the one hand, however also useful for background information.

“The questionnaires [...] take [...] a lot of time, so if they fill them out themselves, it takes them half an hour, 20 minutes. It depends on how well they can concentrate. Some of them can’t fill them out themselves, so I have to do it for them. I read through the questions, and that takes time.” (E001—study nurse)

“[…] of course, I mostly went through the questionnaires with them, depending on how much help they needed. Some were able to fill them out themselves, but many simply needed my assistance. This made it much easier to start a conversation, and I believe I was able to obtain some information that may have been important for the study physician during the consultation, […] information between the lines, what patients simply tell you about their everyday lives. And I think it’s valuable to have that background information.” (E003—study nurse)

#### Perceived benefits of trial participation

Many participants of both the intervention and the control groups reported that they did not feel left alone after discharge by participating in the trial.

“[The fact, that] I was noticed. That I was not forgotten. That is something that is very important in illness.” (I003—patient, intervention group)

Patients and relatives appreciated that the staff took a lot of time for visiting and talking with the patients. Some relatives valued that they were also asked about their health condition during the appointment in the ICU follow-up clinic.

“They (the staff of the follow-up clinic) really took a lot of time. We were given every opportunity and time to talk about our problems. They also explained to us, which was not possible in a normal outpatient clinic, exactly what happened and so on.” (I004—relative, intervention group)

Some participants in the control group even perceived the 6-month follow-up examination as the intervention and perceived that they received more aftercare than expected.

“But now I don’t know, because you said there were two groups, what the other group was. I think I could imagine that I was in a group that does aftercare or something like that. Could that be?” (K002—patient, control group)

### Evaluation of the study context

#### Location of the ICU follow-up clinic

For several participants, the distance of the ICU follow-up clinic from their home was very long (an average of 80 km). Participants of the intervention group were mostly willing to accept the distance of the journey for the 6-month follow-up examination, since they had already received the intervention and rated it positively.

“Let’s just say that somewhere along the line, my inner weaker self says I’m too lazy, I don’t feel like it. But in the end, I was convinced that I would like to go and that I wanted to go.” (I003—patient, intervention group)

“But you say you were happy to accept the distance to visit this outpatient clinic?”

“Yes, definitely, because we felt we were in really good hands down there in general [...]. We were happy to go there.” (I004—relative, intervention group)

However, participants of the control group were more hesitant to make a long journey to the study centre, particularly if there was no specific follow-up care.

“I wouldn’t drive in specifically. I don’t think I would otherwise, either, if it were not COVID-19. So that would be too inconvenient for me to participate in a follow-up survey.” (K002—patient, control group)

One interviewee of the study team suggested that the distance from the place of living to the ICU follow-up clinic should be introduced as an inclusion criterion. Thus, patients living more than 100 km away from the ICU follow-up clinic should be excluded.

“Yes, the distance is certainly a, a point. I’d say 100 kilometres is probably one, a limit that you have to consider as a maximum. Above that, it makes little sense to let people drive here.” (E004—study physician)

#### Professional experience and ICU background as success factors for the ICU follow-up clinic

Both the physicians and the nurses who worked at the ICU follow-up clinic had ICU working experience. Interviewees felt that their professional background was valuable for delivering the follow-up intervention. They were able to answer questions about various issues related to the ICU stay (e.g. delirium).

“Yes, […] delirium, for example, if they [the patients] can remember it, which is rare, thank God, but then to get this feedback again from the ICU staff: That’s okay, that’s normal. That’s how it is.” (E001—study nurse)

#### Challenges in light of organisational matters in the study context

A key organisational aspect was the lack of coding for documenting and billing the services of the ICU follow-up clinic in the corresponding clinical information systems.

“I can perhaps add a small aspect that was a bit difficult at times, namely that [post-intensive] care syndrome did not have a code for diagnosis. In other words, every time we wanted to issue a prescription or something, [...] we practically had to find a different diagnosis for it because we could not use our actual diagnosis.” (E002—study physician)

### Evaluation of the ICU follow-up clinic (intervention)

#### Timing and organisation of the ICU follow-up visit

The study physician scheduled the ICU follow-up visit for 11–12 weeks after discharge from the ICU. Study participants were satisfied with the timing and the length of the follow-up visit.

“We were given every opportunity and time to talk about our problems. They also explained to us in detail, which was not possible in a normal outpatient clinic, including the images and everything, exactly what had happened and so on.”

“So it was really the right time to do it at this interval. And I mean, my husband is now doing really well again, so we’re really very happy so far and I think there would be cases that would perhaps certainly like to do it again later at an interval of four months.” (I004—relative, intervention group)

The participants in the intervention group were each called 2 weeks before their ICU follow-up visit and for the most part could not remember the study at all (field notes). Six (of 13) participants had to postpone the follow-up visit because, for example, they were at the rehabilitation facility longer than planned or still in ongoing hospital treatment (field notes).

One study nurse suggested to organise the process differently and to involve the patients’ relatives more actively.

“Making follow-up calls was a bit of a hassle. Because we tried to get people on board. [...] They should get in touch themselves and be a bit more active. So that the relatives are perhaps more informed and know more about it. So that they can then guide the patients [...] better. And then it might be easier to organize appointments.” (E001—study nurse)

#### Non-utilisation of a support group

Participation in a support group for former ICU patients and their relatives was also offered as part of the follow-up program. This offer was not taken up by any of the study participants. Some people said that they did not need a support group because their need for psychological support was met in another way (e.g. through the support provided by family members). Other participants would have liked to take part in the support group, but the distance of the follow-up clinic from the place of residence was too long to be able to attend an evening appointment in Regensburg.

“But as I live [elsewhere] and would have had to travel so far to the support group, it was too far and the appointment was too late.” (I007—patient, intervention group)

“And then I actually said it would work. If things had turned out differently, I would have said, okay, then we’ll ask for a support group. But we really didn’t need that.” (I001—relative, intervention group)

#### A lot of time for the patients—conflicting goals with regard to time management

The follow-up clinic visit took between one and one-and-a-half hours. This duration was considered lengthy and often impractical in routine clinical practice, where time constraints limit appointment lengths. On the other hand the temporal dimension was found to be of paramount importance for the provision of adequate care, a factor that was highly valued by both staff members and patients.

“So, without the big fuss. And if the patient then wanted to talk, you sometimes had a good hour. So, in terms of time, we probably could have achieved more. So, three patients maximum in the afternoon in the time slot we had. So we always planned very tightly.” (E001—study nurse)

“So we made a lot of home visits. In other words, we sometimes needed three hours for one patient, which would never be feasible in everyday clinical practice.” (E002—study physician)

#### Benefits and perceived effects of the ICU follow-up clinic (patient perspective)

The interviewed patients and relatives saw a benefit to patients from participating in the follow-up clinic program. A decisive factor was the steering function and the specific contact person in the ICU follow-up clinic. Participants had someone who was interested in and cared about the aftercare and drew up a plan alongside them with specific instructions to shape the recovery process. Participants did not feel left alone.

“And that is the good thing that others lack when this study is not available. They are left alone. They hang around there. And you don’t think that a GP here in [our town] knows or realises what you’re [doing] down there. Really. We would be lost. He would have said, keep it up. You can do it. Prescribe pills and off you go.” (I001—patient, intervention group)

Several interviewees described a psychological benefit. They often credited this benefit to the accessibility of a contact person in the follow-up clinic who cared about them. It also played a central role in improving patients’ mental health status that the patients were given enough time to talk about their problems and their care needs were recognised, since the staff had enough experience to assess their situation.

“I would say I had a benefit in the psychological […] area in any case. Because I had a contact person. But even beyond that. That was reassuring, I would say.” (I006—patient, intervention group)

Some participants stated having benefited physically from the ICU follow-up clinic, as their physical limitations were addressed and appropriate referrals to therapists or prescriptions were issued. They often reported they had received referrals for physiotherapy in the ICU follow-up clinic. Some of them noted that they would not have received these referrals from their general practitioners (GPs) because they have a limited budget for medication and physiotherapy and would therefore maybe have experienced a delay in the recovery process. This situation is reflected in the account of a patient from the control group.

“Now, of course, I get physiotherapy and occupational therapy, which is half an hour once a week, 20 minutes. That’s actually nothing. Now I’m actually sitting at home and [...] can’t take part in any rehabilitation programs because I’m not independent enough to walk [...]. You know, I feel a bit left on my own, I have to be honest.” (K004—patient, control group)

Some participants wished to receive care for longer from the follow-up clinic (at least 1 year). Many of the participants wished that the follow-up clinic could be permanently integrated into the care of former ICU patients.

#### Benefits and perceived effects of the ICU follow-up clinic (HCP perspective)

The interviewed HCPs of the study team also recognised the benefits of the participants.

“Simply caring, that is, that someone is there. I think that was the biggest benefit. The second thing was that specific action was assigned. I think the patients appreciate that you do not just say in general, ‘yes, it will get better over time’. We practically tried to draw up a small plan for each patient on what to do next, so that the patients can also set themselves goals and then you can check whether they have achieved them or not”. (E002—study physician)

From the perspective of the interviewed HCPs, the ICU follow-up team also motivated the patients to make use of important treatments and examinations by the GP. Social law queries could also be clarified straight away, as it was possible to contact the social services of the university hospital during the follow-up clinic visit.

According to the interviewees, medication management was also an important component of the follow-up treatment. For some patients, the pain medication was changed, and, according to some interviewees, the medication adherence was higher afterwards, as the prescription of a new medication was done by the ICU follow-up doctor.

“Or they (patients) asked how to proceed with painkillers as well. When can they stop taking painkillers? That makes quite a difference when they take morphine, for example. They are tired, they have changed a bit, they are a bit more lethargic. The information from the GP was apparently not enough.” (E001—study nurse)

The interviewed HCPs also identified a psychosocial benefit for the patients. In some cases, it was enough to take time for a conversation with them.

“The patients were very grateful that we cared about them. It didn’t matter whether we had a lot of suggestions or not. The conversation itself was beneficial for the patients." (E002—study physician)

During the follow-up visit, vital signs were measured, among other things. The interviewees reported that in some cases, the patients were found to have elevated blood pressure, for example, and measures were taken accordingly. In other cases, the ICU follow-up physician became aware of a swallowing disorder and issued prescriptions for speech therapy. One interviewee reported on a follow-up visit in which a patient told of having fallen while in the bathroom a week earlier. Immediately afterwards, another examination was arranged in the clinic to clarify the reasons for the fall.

“Yes, I remember a case where the patient said he fell in the bathroom and was unconscious, and then he was sent to the cardiology outpatient department straight away. And it also came out that there was something wrong with the pacemaker.” (E003—study nurse)

#### Dedicated referral letters for GP colleagues

Some respondents perceived that the GP was overwhelmed with the care of a former ICU patient. For this reason, the cooperation with the follow-up clinic was very important for the patients.

“Because the GP stayed out of it. He said that it looked good. He read the doctor’s reports (from the ICU), but I think he didn’t understand them. I think he was overwhelmed by everything that was going on inside me. He gave me the medication, which was advised by you (ICU follow-up clinic).” (I006—patient, intervention group)

During the 6-month follow-up assessment, one participant in the intervention group mentioned that his GP highly appreciated the information from the referral letter which was issued after the follow-up clinic visit (field notes).

#### Personal benefit for the study team

Both nurses and physicians found a personal benefit in the implementation of the ICU follow-up clinic, since they could accompany the patients’ recovery process. It was important for them to experience that there is a life after the ICU and that it is worth fighting for every patient in everyday life on the ICU.

“That was the personal benefit for me that I see: How are the patients? How do they get out? How long will it take for them to get better? And that was a big relief, that I could see that: Okay, they come back after a year, they gain weight, they get better. […] So, it has brought me a lot. So I would already say that I would do this again from the perspective of an ICU nurse.” (E001—study nurse)

## Discussion

### Summary of key findings

The results relate to the pilot RCT, the study context, and the ICU follow-up clinic itself. Regarding the RCT an overarching result was the high motivation to participate. The inclusion criteria were widely discussed before the study and continue to generate diverse opinions in the evaluation. The offer of home visits for patients with long travel distances to the ICU follow-up clinic was highly appreciated. The main benefits of the ICU follow-up clinic named by participants were being taken seriously and receiving referrals that they would not typically get from a GP, for example. Participants from both the intervention and control groups did not feel left alone due to trial participation. There were even some control group participants that perceived the 6-month follow-up survey as an “intervention.” This, combined with many participants not recalling the informed consent conversation, raises questions about their understanding of the study’s implications and randomisation at the time of inclusion. Regarding the ICU follow-up clinic itself, there was positive feedback from both patients and relatives. Patients reported both physical and psychosocial benefits from participating. Many appreciated having a contact person in the ICU follow-up clinic who cared for them and took plentiful time for the follow-up appointment. Furthermore, the ICU follow-up clinic staff played a crucial role in motivating patients to seek further therapies or make changes to their medication plans. Health professionals valued that the follow-up clinic staff had ICU work experience.

### Strengths and limitations

This study combined data from patients, relatives and healthcare professionals, and therefore explored the experiences with the feasibility study and with the follow-up clinic from three perspectives. We were able to interview more than one third of the total feasibility study sample and both the intervention group and the control group were well represented. We further took field notes and observed parts of the feasibility study to record factors that may not have been captured during the interviews.

Due to the COVID-19 pandemic we were not able to establish the outlined network of outpatient health providers, who provided further treatment to the former ICU patients. For this reason, we did not conduct any interviews with these providers on cooperation or the exchange of information between the ICU follow-up clinic and them in the area of aftercare. Our interviewers were not unconnected to the feasibility study as they were part of the scientific study team. However, they were unrelated to the care provided at the ICU follow-up clinic.

There is a risk of losing or distorting the original meaning in the subsequent translation of the interview data into English. Further, we translated the interviews ourselves, rather than having them professionally translated. Transferability of the findings to other studies and health care contexts is limited, given the sample size and since this study evaluated a specific feasibility study.

There may have been selection bias among the patients who agreed to participate in the RCT. However, all pilot-RCT participants were invited to take part in the qualitative interviews, since we aimed to conduct interviews with 10 participants in the control and the intervention groups, respectively [45]. The interviewer was not involved in the care provided at the ICU follow-up clinic. At the beginning of each interview it was emphasised that the study team was equally interested in identifying both positive and negative experiences. Nevertheless, we acknowledge that social desirability bias can never be completely ruled out in qualitative interviews [54]. Participants from the control group, who did not receive the intervention, also took part in the interviews. To reduce participation barriers, telephone interviews were offered to patients who might otherwise have been unable to participate due to health restrictions.

The transferability of the findings from this study is limited by the complexity of ICU patients, the modest number of interviewees, and the specific context of a single feasibility study. However, all ICU patients (regardless of diagnosis) and their relatives were eligible to participate and all participants were invited without pre-selection. We believe that the findings could provide valuable insights into comparable patient populations, such as post-sepsis or post-COVID survivors, and inform the planning, recruitment and informed consent procedures of similar studies.

### Interpretation

Regarding the inclusion criteria, there were already considerations within the study team during the conduct of the study. Delirium and a minimum duration of ventilation were suggested as further inclusion criteria; these two points may be indicators of a more severe form of PICS [55, 56]. In contrast to the considerations to exclude, e.g. cancer patients from an ICU follow-up clinic, it could also be argued that this offer is far more than aftercare for a specific illness. It seems challenging to define suitable inclusion criteria for the ICU follow-up clinic. On this subject, some of the healthcare professionals we interviewed would support an adjustment to the inclusion criteria for a large-scale study, for example excluding patients who have been ventilated for less than 5 days or those who are already receiving follow-up care due to their underlying disease.

In combination with the large number of participants not actually recalling the informed consent conversation, this might lead to the question whether the study participants were at all able to understand the implications of study participation and randomisation at the time of inclusion. However, this is a problem that often occurs in RCTs, as shown in a 2015 meta-analysis [57]. Despite feeling well informed, several patients and relatives could not remember the informed consent discussion. Few remembered details of randomisation or their allocated group. The conversation may have taken place too early, as many did not feel able to understand the implications of the trial when they were discharged from the ICU. Only one person commented on their acceptance of randomisation. The various challenges associated with the provision of informed consent for randomised trials have been the subject of research for many years [58, 59]. A recent review of the proportion of clinical trial participants who were aware of the components of informed consent revealed that the majority of participants understood fundamental components (e.g. voluntary participation), but not randomisation [60]. An effectiveness trial of an ICU follow-up clinic should carefully consider the timing of consent, use simple explanations, and include procedures to ensure that participants and their families understand the implications of their participation in the study, e.g. a two-stage procedure: possible participants could be initially approached by a study physician and approached again 1 day later by a study nurse to explain practical aspects of the study in more detail and to answer remaining questions. In addition, a reasonable balance should be found between two important aspects: the accessibility of potential study participants (i.e. they should not be lost due to transfer) and their capacity to consent (i.e. they should already be able to understand the information about study participation and make an informed decision).

The high demand for follow-up care for former ICU patients was reflected in the high level of participation and acceptance of our study and the statements regarding the motivation to participate.

In addition, it may also indicate that among ICU patients the need for follow-up is so great that even the 6-month follow-up survey was interpreted as “the intervention” by some participants in the control group. This could be because they are not receiving any other form of follow-up care.

Participants from both the intervention and the control groups did not feel left alone as a result of trial participation. There is also evidence that simply completing questionnaires can have an impact on perceived health condition [61]. It is noteworthy that such a low-threshold offer has already been positively evaluated by patients. This suggests that simply caring for and having access to individuals who were willing to listen to the needs of former ICU patients can already have a beneficial impact.

In relation to our findings in the quantitative analysis where 85% of participants of the feasibility study completed the questionnaires with assistance [40], some interviewees of the process evaluation study indicated not having been bothered with the questionnaires, while others complained about the length or were not able to read the amount of documents. Further, both study nurses mentioned that participants completed questionnaires with their assistance. We yet assume that the participants accepted the support in completing the questionnaires because a member of the study team was present at the time and was able to provide support. Given that the time allowed was more generous than in standard care, but still limited, we assume that participants simply accepted this assistance. From the evaluation presented here, we cannot conclude that there were essential difficulties with the scope and comprehensibility of the measurement instruments used. However, we did not perform individual tests on the respective measuring instruments (e.g. SF-12, PHQ-D, PTSS-10).

Since for several participants the distance of the ICU follow-up clinic from their home was very long, those patients who could not drive to the follow-up clinic were offered a home visit. These patients were grateful for this opportunity. However, this option is difficult to implement in a larger trial or even in everyday clinical practice. While home visits are highly valued by patients, they are resource-intensive and may increase costs and staffing demands in a larger trial or routine implementation. Considering the distance between the place of living and the ICU follow-up clinic, this could also serve as an inclusion criterion for a later effectiveness trial.

It also appeared helpful that the ICU follow-up clinic staff took on a steering function and motivated patients to seek further therapies or made changes to the drug plan. It was considered important by the health professionals that the staff of the ICU follow-up clinic had working experience on the ICU. This means that staff specifically with ICU experience should be deployed in an ICU follow-up clinic [62, 63], regardless of which clinical pictures are presented on the basis of PICS and where the ICU follow-up clinic is located organisationally in a facility.

The ICU follow-up clinic received positive feedback from both patients and relatives. Some patients reported a physical, others a psychosocial benefit from visiting the ICU follow-up clinic. Many of them were very positive about the accessibility of a contact person in the ICU follow-up clinic who cared for them and took a lot of time to carry out the follow-up appointment. It seems that our participants benefited most from having had a contact person and coordinator in the ICU follow-up clinic. Patients and relatives highlighted that the ICU follow-up team took time to talk with them about their health status, drew up a plan with instructions, made changes to the medication plan and interacted with their GP if necessary. Our findings are consistent with those of McPeake et al., who showed that patients valued active listening and ongoing referral to other services. They also highlighted the need for a care coordinator who is able to link acute practice with GPs [63]. Attending the ICU follow-up clinic gave the intervention-group participants reassurance about the course of recovery. Other authors also noted that expert reassurance is extremely important for former ICU patients [26, 63]. Support was provided to the intervention group in various ways: offering a detailed medical consultation, performing tests and measuring vital signs, but also in the form of referrals to other specialists, such as physiotherapists. Prinjha et al. stated that being referred to other specialists gave participants a sense of security, knowing their problem was being addressed and they would receive further care [26].

Our study showed that it is important for the ICU staff to follow ICU patients’ recovery process and thus to get feedback not only about the time and care on the ICU itself, but also about the life after ICU. We stated, like Flinterud et al. before, that ICU follow-up was found to increase the staff’s motivation to continue working with critically ill patients [64], since ICU follow-up programs also offer the opportunity to see positive outcomes of challenging cases [62]. This has the positive effect that an experienced and competent staff is able to offer the patients appropriate care, but also that the staff itself benefits from the ICU follow-up clinic.

There is a qualitative systematic review ongoing on experiences of ICU survivors attending ICU follow-up services [65]. In their protocol, Delaney et al. outlined that a limited understanding of the patient experience when attending follow-up services might contribute to the fact that effectiveness trials on ICU follow-up services showed no or only less-than-expected improvement in HRQOL, PTSD or mortality [20, 66].

The question therefore arises as to what extent the singular (quantitatively measured) outcome of health-related quality of life chosen by us and other research groups should be the primary endpoint of the effect of the ICU follow-up clinic. Further, it might be considered how long the follow-up period should be after discharge from the ICU. Evidence suggests that 12 months may not be long enough to reliably measure effects with the available instruments, particularly in terms of quality of life [67]. The follow-up period of 6 months chosen in our pilot study seems clearly too short in this context.

A recent systematic review of 71 publications [43] addressed this point, showing that only 30% of studies observed for more than 6 months. The review also showed that most post-ICU programs are not developed systematically and rarely use program-logical models. Furthermore, little fidelity data is collected in the areas of training, delivery, receipt and enactment (only 19% use a framework and 40% use detailed fidelity measurement). By contrast, our ICU follow-up program has been consistently developed in accordance with the MRC framework. We created a comprehensive logic model [40, 45] and iteratively refined the ICU follow-up clinic concept with relevant stakeholder groups, including patients, relatives, ICU clinicians, general practitioners, and therapists. This approach was found in only 11% of the studies described in the review. Furthermore, we quantitatively and qualitatively monitored the entire study process continuously, and surveyed acceptance among relatives and clinicians. This was only the case in 8/71 and 2/71 of the studies in the review, respectively.

The benefits we identified in our process analyses as noteworthy, namely feeling taken seriously and getting referrals patients do not get from a GP, are not represented by HRQOL or functioning-measurement instruments and it could be that measuring effects primarily in relation to HRQOL falls short [68].

In the German context, however, the following should be noted: in order for an intervention such as this ICU follow-up clinic to be incorporated into standard care and covered by statutory health insurance, it is necessary for a study to demonstrate its benefits. The German Social Code stipulates HRQOL as an important outcome for those studies [69].

In the context of choosing appropriate outcomes for studies on ICU follow-up clinics, it also seems desirable to consider a core outcome set for ICU survivorship research [70], which could be developed or adapted from existing sets in the field of intensive care medicine [71, 72].

## Conclusions

The participants of this process evaluation considered ICU follow-up to be a highly relevant aspect of post-intensive care. The interviews revealed that the ICU follow-up clinic addressed important needs of former ICU patients and their relatives. The findings further support our conclusions from the feasibility study that our concept of a pragmatic randomised controlled trial on the effects of an ICU follow-up clinic was considered acceptable and viable across the stakeholder groups (patients, relatives, healthcare professionals). Although we cannot use this study to draw conclusions about the outcomes of the ICU follow-up clinic, our findings indicate that intervention components were applied that met patients’ needs. A large-scale study appears to be feasible.

## Supplementary Information

Supplementary Material 1: Interview guides.

## Data Availability

Transcripts will not be shared in their entirety to protect the participants. However, requests to the corresponding author for extracts of data will be considered on a reasonable individual basis.

## Abbreviations

GP: General practitioner
HCP: Healthcare professional(s)
HRQOL: Health-related quality of life
ICU: Intensive care unit
PICS: Post-intensive care syndrome
RCT: Randomised controlled trial
SOFA: Sequential organ failure assessment
