# Supplementary material for: Piloting an ICU follow-up clinic — qualitative process evaluation alongside a feasibility study
Source: Pilot Feasibility Stud. 2026 May 9;12:61. doi: 10.1186/s40814-026-01827-5 (PMC13156872; doi:10.1186/s40814-026-01827-5)
Supplement: Supplementary file 1 — Supplementary Material 1: Interview guides. [file 40814_2026_1827_MOESM1_ESM.pdf]

Interview guide patients intervention group

Instruction: I would first like to ask you a few questions about the study. By study I mean, for example, that all participants were in two different groups, that we collected clinical data from you, that you completed questionnaires and that there will be a scientific evaluation at the end. *Prompts in italics.*

Evaluation of the study	**Acceptance**<br>1. As a participant in the intervention group, what do you think of the study?<br>2. How did you feel about participating in the study? *What was easy for you, what was difficult? What was unusual?*<br>**Consent**<br>3. When did you consent to the study and why at that time? *Immediately after the informed consent form or later (after a discussion with the relatives)*?<br>**Acceptance of randomisation**<br>4. How well were you able to accept the random allocation to one of the two groups?<br>**Questionnaires**<br>5. Can you name any positive or negative aspects of the questionnaires used (baseline, follow-up)?<br>**Information on the study**<br>6. How would you rate the information provided by the physcian about the study?<br>7. How would you rate the amount of information you received about the study and the upcoming treatment in the ICU follow-up clinic? *If not enough: What additional information would you have liked?*<br>**Treatment effects on areas of life**<br>8. In which areas of your life have you benefited most from the treatment and in which less - *physically or mentally?*<br>**Challenges and concerns**<br>9. Do you recognise any difficulties or problems with the study?

Instruction: I would now like to ask you some questions about the context of the study and the aftercare clinic in the intensive care unit. The questions mainly relate to organisational and practical aspects of the aftercare clinic in the intensive care unit.

Evaluation of the context	**Health restrictions**<br>10. To what extent did your state of health restrict you when you had to come to the university hospital for the examinations?<br>**Organisation aftercare clinic**<br>11. How do you rate the organisation of the follow-up clinic with regard to the location at the university hospital and the opening hours? *(More) suitable opening hours? Suggestions for the location?*<br>**Distance home-clinic**<br>12. How far did you have to travel to attend your appointment at the follow-up clinic?<br>13. What is the maximum distance you would be willing to travel?<br>**6-month follow-up examination**<br>14. What do you think of the fact that you had to come to the university hospital several times to take part in a study?

Instruction: Then I would like to ask you a few more questions about the treatment in the ICU follow-up clinic. Our aim is to evaluate this intervention and identify starting points for future improvements.

Evaluation of the intervention	**Scheduling for the visit**<br>15. What do you think of the physician setting the date for the visit? *Would you have liked to have made the appointment yourself and would this have been possible for you?*<br>16. How many weeks before the visit were you back home?<br>17. Was the timing of the visit right, too early or too late for you?<br>**The visit to the ICU follow-up clinic itself**<br>• **Satisfaction**<br>18. To what extent were you satisfied with your visit to the follow-up clinic? *What was positive, what was negative? Were you able to express your own concerns?*<br>• **Effects of the treatment**<br>19. How well did you benefit from the additional treatment during the consultation?<br>• **Components**<br>20. How do you rate the individual components and the process of the follow-up clinic? *Was anything missing or unnecessary for you?*<br>• **Physician-patient relationship**<br>21. Did you already know the physician in the follow-up clinic from the intensive care unit? *If so, was that helpful?*<br>• **Referral to therapists**<br>22. How did you perceive the referral to therapists during counselling? *Helpful, time-consuming, overwhelming, understandable?*<br>• **Temporal aspects**<br>23. How do you rate the follow-up clinic in terms of the duration of the appointment, the number of appointments (*more than 1 appointment?*) or the limitation of the continued care to six months (*too short/long, appropriate*)?<br>**Monitoring by telephone**<br>24. How do you rate the calls from the nursing staff or physicians? *Helpful, unnecessary?*<br>**Self-help groups for patients**<br>25. What do you think of self-help groups?<br>26. What reasons did you have for not participating?<br>**Integration of treatment**<br>27. To what extent did the ICU follow-up clinic supplement or replace other medical appointments?<br>28. To what extent did the recommendations of the various providers agree or contradict each other?<br>**Home visits (if applicable)**<br>29. What was it like for you to have the physician and nurse come to your home?

Interview guide patients control group

Instruction: To begin with, I would like to ask you a few questions about the study. By study I mean, for example, that all participants were in two different groups, that we collected clinical data from you, that you completed questionnaires and that there will be a scientific evaluation at the end.

Evaluation of the study	**Acceptance**<br>1. As a participant in the control group, what do you think about the study?<br>2. How did you feel about participating in the study? *What was easy for you, what was difficult? What was unusual?*<br>**Consent**<br>3. When did you consent to the study and why at that time? *Directly after the informed consent or later (after talking to a relative)?*<br>**Acceptance of randomisation**<br>4. How well were you able to accept the random allocation to one of the two groups?<br>**Discrimination**<br>5. Did you consider withdrawing from the study during the course of the study because you were assigned to the control group? *Why*?<br>6. During the study, did you also wish to be cared for in the ICU follow-up clinic? *If so,* why? *Can you explain this wish?*<br>**Questionnaires**<br>7. What do you think about the questionnaires used (*baseline, follow-up*)?<br>8. What positive or negative aspects can you say about the questionnaires?<br>**Information on the study**<br>9. How would you rate the information provided by the physician about the study?<br>10. What do you think of the amount of information you received about the study? *If not enough: What additional information would you have liked to have received?*<br>**Challenges and Concerns**<br>11. What difficulties or problems do you see with the study?

Introduction: I would now like to ask you a few questions about the context of the study and the ICU follow-up clinic. The questions mainly relate to organisational and practical aspects of the study.

Evaluation of the context	**Distance home-clinic**<br>12. Assuming you had the opportunity to make an appointment at an ICU follow-up clinic, what is the maximum distance you would be prepared to travel to attend this appointment?<br>**6-months follow-up examination**<br>13. What do you think about the fact that you had to come to the university hospital to take part in a study (if 6-months follow-up was onsite)/ would have to come (if follow-up was by telephone)?

Admission: I would then like to ask a few more questions relating to treatment in the intensive aftercare outpatient clinic.

Evaluation Intervention	**Utilisation of offer**<br>14. Can you imagine using the services of an ICU follow-up clinic on a voluntary basis? *If so, under what conditions?*

Interview guide relatives intervention group

Instruction: To begin with, I would like to ask you a few questions about the study in which your relative took part. By study, I mean, for example, that there were two different groups, we collected clinical data from your relative, they completed questionnaires and there is a scientific evaluation at the end.

Evaluation study	**Decision on study participation**<br>1. Did your relative involve you in the decision to participate?<br>2. How did you advise your relative about participating in the study?<br>**Information**<br>3. How would you rate the information provided to your relative about the study before consent was given?<br>4. How would you rate the amount of information your relative received about the study and the upcoming treatment in the ICU follow-up clinic?<br>**Acceptance of randomisation**<br>5. What do you think of the randomised group assignment?<br>**Concerns**<br>6. What difficulties or problems do you see with the study?<br>7. How do you feel about the study after the continued care?

Introduction: I would now like to ask you a few questions about the context of the study and the ICU follow-up clinic. The questions mainly relate to organisational and practical aspects of the clinic.

Evaluation context	**Accompanying the patient**<br>8. Were you prepared to accompany the patient to the appointment at the university hospital? *Why*? (*Interest, own clarification of questions, health problems?*)<br>**Distance home-clinic**<br>9. What is the maximum distance you would accept to accompany the patient to the appointments of an ICU follow-up clinic?<br>**Organisation**<br>10. What do you think about the organisation of the clinic in terms of location and opening hours?

Introduction: Now I would like to ask a few more questions relating to treatment in the ICU follow-up clinic. Our aim is to evaluate this intervention and identify starting points for future improvements.

Evaluation Intervention	**ICU follow-up clinic visit**<br>• **Satisfaction**<br>11. To what extent were you satisfied with your visit to the clinic? *What was positive, what was negative*? *Were you able to express your own concerns?*<br>• **Treatment effect**<br>12. How well did you and your relative benefit from the additional treatment during the consultation?<br>• **Temporal aspects**<br>13. How would you rate the organisation of the clinic in terms of duration of the appointment, the number of appointments (*more than 1 appointment?*) or the limitation of the continued care to six months (*too short/long, appropriate*)?<br>**Self-help groups**<br>14. What do you think about the services offered by the self-help groups?<br>15. What reasons do you have for not participating?

Interview guide relatives control group

Instruction: To begin with, I would like to ask you a few questions about the study in which your relative took part. By study, I mean, for example, that there were two different groups, we collected clinical data from your relative, they completed questionnaires and there is a scientific evaluation at the end.

Evaluation study	**Decision on study participation**<br>1. Did your relative involve you in the decision to participate?<br>2. How did you advise your relative about participating in the study?<br>**Information**<br>3. How would you rate the information provided to your relative about the study before consent was given?<br>4. How would you rate the amount of information your relative has received?<br>**Acceptance of randomisation**<br>5. What do you think of the randomised group assignment?<br>6. During the course of the study, did you wish that your relative was also in the treatment group for additional support?<br>**Concerns**<br>7. What difficulties or problems do you see with the study?

Introduction: I would now like to ask you a few questions about the context of the study and the ICU follow-up clinic. The questions mainly relate to organisational and practical aspects of the clinic.

Evaluation context	**Distance home-clinic**<br>8. What is the maximum distance you would accept to accompany your relatives to appointments at an ICU follow-up clinic?<br>**Discrimination**<br>9. How do you feel when you think about the group membership of your relatives?

Interview guide physicians and nurses

Introduction: To begin with, I would like to ask you a few questions about the study itself.

Evaluation study	**Resources**<br>1. How do you rate the available financial and human resources for the corresponding implementation of the study?<br>2. Which aspects do you see as beneficial in the context of the study?<br>3. How should a study team be organised in order to conduct a large-scale RCT efficiently?<br>**Recruitment**<br>4. How do you rate the screening and recruitment of patients?<br>5. How much time and resources did the patient screening take?<br>6. What difficulties arose and in which areas had adjustments to be made?<br>7. With regard to the establishment of several outpatient clinics, do you consider the inclusion criteria of the study to be suitable? *To what extent do the inclusion criteria cover all patients who require follow-up care*?<br>8. Should the same inclusion criteria be used for a large-scale RCT? What speaks in favour, what against?<br>**Realisation**<br>9. Thinking back to the entire implementation of the study, how do you rate it?<br>10. How do you assess the feasibility of an ICU follow-up clinic on the basis of the study conducted? *(Organisation, effort, effect, feedback from patients & relatives?)*<br>11. What difficulties and challenges arose in the course of the study?<br>12. How can these difficulties be overcome with regard to a large-scale RCT?

Instruction: In the following, I would be interested to know how you assess the context of the study and the intervention with regard to organisational aspects, for example.

Evaluation context	**Co-operation**<br>13. How did the cooperation between you and the nursing staff/you and the physicians in the ICU follow-up clinic work?<br>14. How did the co-operation between the clinical team and the scientific study team work?<br>15. How did the co-operation with the nursing staff in the outpatient pneumology department work (*with regard to the measurement of vital signs*)?<br>**Organisation**<br>16. How did the shift planning work? What was easy, what was difficult?<br>17. What was the experience with the premises of the outpatient clinic? Were they appropriate and available for the ICU follow-up clinic?<br>18. To what extent are two physicians sufficient to run the ICU follow-up clinic?<br>19. Were there any cancelled appointments due to staff shortages?<br>**Forwarding**<br>20. How did the referral of patients to outpatient therapists work?<br>21. How did the outpatient therapists react to the referral?<br>**Physician-patient relationship**<br>22. To what extent does the prior knowledge of the patient influence your behaviour during the study? (*Informative discussion: preparation time, ...?*)<br>**Taking on a role**<br>23. Physicians: What was it like for you to switch between the role of study physician in terms of recruitment and information and the role as a practitioner in the ICU follow-up clinic during the course of the study?<br>24. Nursing: What was it like for you to switch between the role of study nurse with regard to screening, recruitment and telephone reminders and the role of nursing staff in the ICU follow-up clinic?

Referral: Finally, I would like to ask you a few questions about the intervention in the ICU follow-up clinic.

Evaluation Intervention	**ICU follow-up clinic visit**<br>• **Benefit**<br>25. What do you think the patients and their relatives benefited most from during the visit?<br>• **Compliance and acceptance**<br>26. How do patients express their interest, motivation and co-operation? *"Compulsory appointment" vs. "support programme"?*<br>27. How do you rate patient acceptance of physical examinations? Should these measurements be used further on?<br>• **Physician's letter**<br>28. How much time did it take on average to write the physician's letter?<br>29. To what extent did the patients want to discuss the physician's letter together?<br>• **Potential for improvement**<br>30. In what areas could the ICU follow-up clinic visit process be improved?<br>31. To what extent would processes be variable in terms of time?<br>**Telephone contacts**<br>32. How much time did the telephone contact attempts take?<br>33. What was the patient's reaction to the call from the physician/nurse?<br>34. To what extent should the telephone contacts be continued if an ICU follow-up clinic were to be widely offered?<br>**Self-help groups**<br>35. How was the organisation of the self-help groups in terms of time and premises?<br>36. Who should best take over the management of the groups?
